# Curcumin: Total-Scale Analysis of the Scientific Literature

**DOI:** 10.3390/molecules24071393

**Published:** 2019-04-09

**Authors:** Andy Wai Kan Yeung, Michal Horbańczuk, Nikolay T. Tzvetkov, Andrei Mocan, Simone Carradori, Filippo Maggi, Joanna Marchewka, Stefania Sut, Stefano Dall’Acqua, Ren-You Gan, Lyubka P. Tancheva, Timea Polgar, Ioana Berindan-Neagoe, Vasil Pirgozliev, Karel Šmejkal, Atanas G. Atanasov

**Affiliations:** 1 Oral and Maxillofacial Radiology, Applied Oral Sciences, Faculty of Dentistry, The University of Hong Kong, Hong Kong, China; 2 Warsaw University of Life Sciences, Faculty of Applied Informatics and Mathematics, 02-787 Warsaw, Poland; mifune6@gmail.com; 3 Institute of Molecular Biology “Roumen Tsanev”, Department of Biochemical Pharmacology and Drug Design, Bulgarian Academy of Sciences, Acad. G. Bonchev Str., Bl. 21, 1113 Sofia, Bulgaria; ntzvetkov@gmx.de; 4 Pharmaceutical Institute, University of Bonn, An der Immenburg 4, 53121 Bonn, Germany; 5 Department of Pharmaceutical Botany, “Iuliu Haţieganu” University of Medicine and Pharmacy, 23 Ghe. Marinescu Street, 400337 Cluj-Napoca, Romania; mocan.andrei@umfcluj.ro; 6 Laboratory of Chromatography, Institute of Advanced Horticulture Research of Transylvania, University of Agricultural Sciences and Veterinary Medicine, 400372 Cluj-Napoca, Romania; 7 Department of Pharmacy, University “G. d’Annunzio” of Chieti-Pescara, Via dei Vestini 31,66100 Chieti, Italy; simone.carradori@unich.it; 8 School of Pharmacy, University of Camerino, 62032 Camerino, Italy; filippo.maggi@unicam.it; 9 The Institute of Genetics and Animal Breeding, Polish Academy of Sciences, Jastrzębiec, 05-552 Magdalenka, Poland; J.Marchewka@ighz.pl; 10 Department of Agronomy, Food, Natural Resources, Animals and Environment (DAFNAE), Agripolis Campus, University of Padova, 35020 Padova, Italy; stefania_sut@hotmail.it; 11 Department of Pharmaceutical and Pharmacological Sciences University of Padova, 35020 Padova, Italy; stefano.dallacqua@unipd.it; 12 Department of Food Science & Technology, School of Agriculture and Biology, Shanghai Jiao Tong University, Shanghai 200240, China; renyougan@sjtu.edu.cn; 13 Department of Behavioral Neurobiology, Institute of Neurobiology, Bulgarian Academy of Sciences, 1000 Sofia, Bulgaria; lyubkatancheva@gmail.com; 14 GLOBE Program Association (GLOBE-PA), Grandville, MI, USA; timea.polgar@envisionbiotechnology.com; 15 MEDFUTURE - Research Center for Advanced Medicine, 400037 Cluj-Napoca, Romania; ioananeagoe29@gmail.com; 16 Research Center for Functional Genomics, Biomedicine and Translational Medicine, Institute of Doctoral Studies, “Iuliu Hatieganu” University of Medicine and Pharmacy, 400037 Cluj-Napoca, Romania; 17 Department of Experimental Pathology, “Prof. Dr. Ion Chiricuta”, The Oncology Institute, 400037 Cluj-Napoca, Romania; 18 The National Institute of Poultry Husbandry, Harper Adams University, Shropshire TF10 8NB, UK; vpirgozliev@harper-adams.ac.uk; 19 Department of Natural Drugs, Faculty of Pharmacy, University of Veterinary and Pharmaceutical Sciences Brno, Palackého tř. 1946/1, 612 42 Brno, Czech Republic; karel.mejkal@post.cz; 20 Department of Pharmacognosy, University of Vienna, 1090 Vienna, Austria

**Keywords:** curcumin, pharmacology, bibliometrics, biochemistry, cancer, citation analysis, VOSviewer, Web of Science

## Abstract

The current study aimed to provide a comprehensive bibliometric overview of the literature on curcumin, complementing the previous reviews and meta-analyses on its potential health benefits. Bibliometric data for the current analysis were extracted from the Web of Science Core Collection database, using the search string TOPIC=(“curcumin*”), and analyzed by the VOSviewer software. The search yielded 18,036 manuscripts. The ratio of original articles to reviews was 10.4:1. More than half of the papers have been published since 2014. The major contributing countries were the United States, China, India, Japan, and South Korea. These publications were mainly published in journals representing the following scientific disciplines: biochemistry, chemistry, oncology, and pharmacology. There was a significant positive correlation between the total publication count and averaged citations per manuscript for affiliations, but not for countries/regions and journals. Chemicals that were frequently mentioned in the keywords of evaluated curcumin publications included curcuminoids, resveratrol, chitosan, flavonoids, quercetin, and polyphenols. The literature mainly focused on curcumin’s effects against cancer, inflammation, and oxidative stress. Cancer types most frequently investigated were breast, colon, colorectal, pancreatic, and prostate cancers.

## 1. Introduction

Turmeric (*Curcuma longa* L., Zingiberaceae) is traditionally used in Indian medicine for the treatment of various illnesses, mainly connected with inflammatory processes. According to the Plant List database (http://www.theplantlist.org), the *Curcuma* genus includes more than 93 species, with different use and content of active substances. Turmeric is cultivated mainly in India, China, Indonesia, Jamaica, and Peru. The medicinal applications of turmeric have been known for thousands of years, especially by Ayurvedic therapy, which uses turmeric for stomach disorders, as a tonic, for blood cleansing, as well as for prevention or treatment of skin diseases. In addition, an administration of turmeric is recommended in disorders of bile production, anorexia, rhinitis, sinusitis, cough, diabetic lesions, disorders of liver functions, and rheumatism. The contemporary focus on turmeric and its substances can be tracked back to the 1970s, when mechanisms of its anti-inflammatory, antibacterial, and antioxidant properties started to be evaluated. However, readers should note that most of the scientific papers reporting the biological effects of curcumin represent preliminary in vitro data, obtained with biochemical (cell-free) assays or on cell culture models.

The aroma of turmeric is based on a bunch of sesquiterpenes. Typical examples are *(S)-ar*-turmerone, zingiberene, β-turmerone, and curlone. Their ratio and the presence of a wide range of other volatile compounds (e.g., monoterpenes) in various turmeric cultivars affect the aroma of curcuma when used as a food seasoning [1]. The marker substances isolated from turmeric are called curcuminoids. The main representative of this group of compounds is the *bis*-α,β-unsaturated diketone curcumin (diferuloylmethane). Its structure had already been described by 1910. Curcumin is a compound showing keto-enol tautomerism, with the predominance of the keto form in acidic environment and stable enol form under basic conditions. Turmeric contains 2–5% of curcumin, according to the origin. After extraction, it is characterized as a yellow crystalline powder, practically insoluble in water, showing good solubility in fats and ethanol [2].

Curcumin is famous for its potential applications in the prevention and treatment of cancer [3], as well as for its anti-inflammatory, antioxidant [4], and antiangiogenic activities [5]. These activities were potentiated by the design and synthesis of structurally related analogues following a “lead optimization” approach. Meanwhile, curcumin may also inhibit β-amyloid formation and hence be potentially useful for preventing and treating Alzheimer’s disease [6,7,8,9,10]. There are several highly cited reviews that summarize the above-mentioned important research findings about the health beneficial effects of curcumin [11,12]. Meanwhile, similar to those in other research fields, such as in nutritional neurosciences [13,14,15,16], there have already been many meta-analyses of the effects of curcumin, including those with focus on downregulation of human tumor necrosis factor (TNF)-alpha levels [17], alleviation of joint arthritis [18], and regulation of blood lipid levels [19]. With so many publications available in the existing literature, a bibliometric analysis could identify and quantitatively analyze the major themes of the curcumin research literature, as well as summarize the citation performance of various contributors and topics. Similar analyses have already been published in other research fields such as ethnopharmacology [20], food sciences [21], neuropharmacology [22], nutraceuticals [23], and oncology [24].

The current study aimed to identify and analyze publications on curcumin to outline the major contributors in terms of author affiliations, countries/regions, and journals. It also aimed to reveal the major research themes present in the literature about curcumin, based on the publication and citation data. These pieces of information should be helpful for readers, including audience without deep previous knowledge of the topic, to quickly have a general overview of the curcumin literature landscape, including prominent authors, major output countries, and prevailing major research topics and trends. The provided information can also be used to identify potentially promising research directions and possible collaboration partners, and to give initial orientation to direct further more in-depth searches for the identification of relevant publications or research opportunities.

## 2. Materials and Methods

In November 2018, we accessed the Clarivate Analytics-owned Web of Science (WoS) Core Collection online database to identify curcumin publications with the following search string: TOPIC=(“curcumin*”). This search string identified publications that mentioned the word “curcumin” or its derivatives in the title, abstract, or keywords. We did not place additional restriction on the search strategy, such as publication year, publication type, or language. To gain insides into research focused on extracts of the plant *Curcuma longa*/turmeric, an additional analysis was performed to evaluate the publications that mentioned *Curcuma longa* or turmeric without mentioning curcumin, using the search strategy: TOPIC=(“curcum*” OR “tumeric*” OR “turmeric*” NOT “curcumin*”).

### Data Extraction

The publications identified from the search were evaluated by: (1) publication year; (2) author affiliations; (3) countries/regions of the affiliations; (4) journal title; (5) WoS category; (6) publication type; (7) language; and (8) total citation count. The full records and cited references of these publications were downloaded and loaded into VOSviewer for further bibliometric analyses.

The VOSviewer software (v.1.6.8, 2018) is capable of extracting and analyzing the semantic contents of the titles, abstracts, and keywords of publications, relating them to the citation count data and generating a bubble map to visualize the results [25]. Default parameters were used for the analyses and creation of bubble maps. The font size of the words in the bubble map indicates their frequency of occurrence (multiple appearances in a single publication count as one). Two words are nearer to each other if they co-occurred in the evaluated publications more frequently. Only words that appeared in at least 1.0% (*n* = 181) of the manuscripts were analyzed and visualized. For the keyword map, full counting method was used, meaning that each co-occurrence link carried the same weight. The default “association strength method” was used for normalization of the co-occurrence matrix with default values of attraction and repulsion.

We tested the possible correlation between total publication count and averaged citations per manuscript for the affiliations, countries/regions, and journals. For these data, we only considered entities that contributed to at least 0.01% (*n* = 19) of the publications. Pearson’s correlation test was performed in SPSS 25.0 (IBM, New York, NY, USA). Test results with *p* < 0.05 were considered statistically significant.

## 3. Results

The primary literature search resulted in 18,036 publications. The earliest articles on curcumin indexed in WoS were published in 1970 and 1971, and they investigated the hypocholesterolemic effect of curcumin in rats [26,27]. More than half of the analyzed papers have been published since the year 2014. The large number of publications since 2014 could be attributed to the increased publication productivity of China (publications since 2014 = 2443; 68.9% of total contributions) and India (publications since 2014 = 1620; 51.8% of total contributions). The numbers of original articles (*n* = 14,315) and reviews (*n* = 1378) were in the ratio of 10.4:1. The majority of the publications were written in English (*n* = 17,871, 99.1%). Contributions came from 7729 organizations (author affiliations) located in 125 countries/territories and were published in 2905 journals. The top five WoS categories of the manuscripts were pharmacology and pharmacy (*n* = 3590, 19.9%), biochemistry and molecular biology (*n* = 2526, 14.0%), oncology (*n* = 1,894, 10.5%), multidisciplinary chemistry (*n* = 1485, 8.2%), and medicinal chemistry (*n* = 1469, 8.1%). The top five contributors with regard to journal, organization, and country/territory are listed in Table 1. The five most productive countries were from Asia, except the United States.

**Figure 1:**
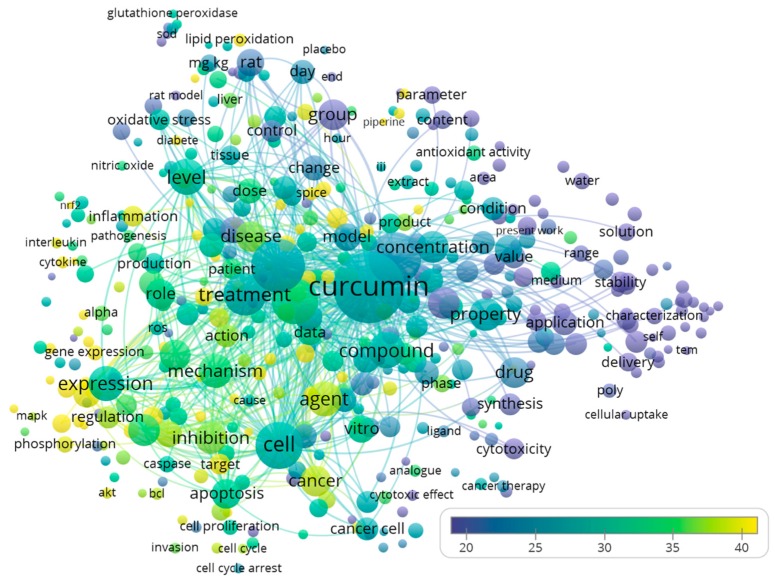
Bubble map visualizing words from titles and abstracts of the 18,036 curcumin publications. We used VOSviewer software to analyze and visualize recurring terms from titles and abstracts. Only words that appeared in at least 1.0% (*n* = 181) of the publications were analyzed and visualized. There were 422 terms that appeared in at least 1.0% of the evaluated publications. The word size indicates the appearance frequency of the words (multiple appearances in a single manuscript count as one). Two words are closer to each other if they co-occurred more frequently in the evaluated publications. Bubble colors represent the averaged citations of the terms.

There were 422 terms that appeared in at least 1.0% (*n* = 181) of the evaluated publications. By analyzing these words in the titles and abstracts of the 18,036 publications, we found that some notable highly cited themes of the publications were related to the effects of curcumin and its derivatives against cancer (*n* = 2583, citations per publication = 37.8), inflammation (*n* = 1210, citations per publication = 38.8), and oxidative stress (*n* = 1266, citations per publication = 29.6) (Figure 1). The top 20 recurring terms are listed in Table 2.

Moreover, we examined the data to identify the prevalence of the use of metabolomics, proteomics, genomics, and transcriptomics, as well as clinical trials in curcumin research. “Metabolomic(s)” was mentioned in 42 publications, with a total of 293 citations. For example, it was found that, via nuclear magnetic resonance (NMR) spectroscopy-based metabolomics, in breast cancer cells the major target of curcumin was metabolism of glutathione [28]. At the same time, “proteomic(s)” was mentioned in 62 publications, with a total of 526 citations. An example is a study showing that curcumin could bind to 197 proteins in HCT116 colon cancer cell line, which results in downregulation of cellular protein synthesis and induction of autophagy [29]. Meanwhile, “genomic(s)” was mentioned in 77 publications, with a total of 1,691 citations. A representative study revealed that 1α,25-dihydroxyvitamin D(3) and curcuminoids had additive effects in stimulating amyloid clearance in patients of Alzheimer’s disease [30]. Similarly, the term “transcriptomic(s)” was mentioned in 20 publications, with a total of 244 citations. For instance, it was found that curcumin-treated lipopolysaccharide (LPS)-primed microglia showed limited neurotoxicity with the decrease of apoptosis [31]. In terms of original articles with the term “clinical trial*”, there were 691 publications, with a total of 27,922 citations. Exemplars included Phase I clinical trial of oral curcumin (C3 complex) intake in patients with advanced colorectal cancer refractory to standard chemotherapies [32]; and Phase II trial of oral curcumin intake in patients with advanced pancreatic cancer [33].

**Figure 2:**
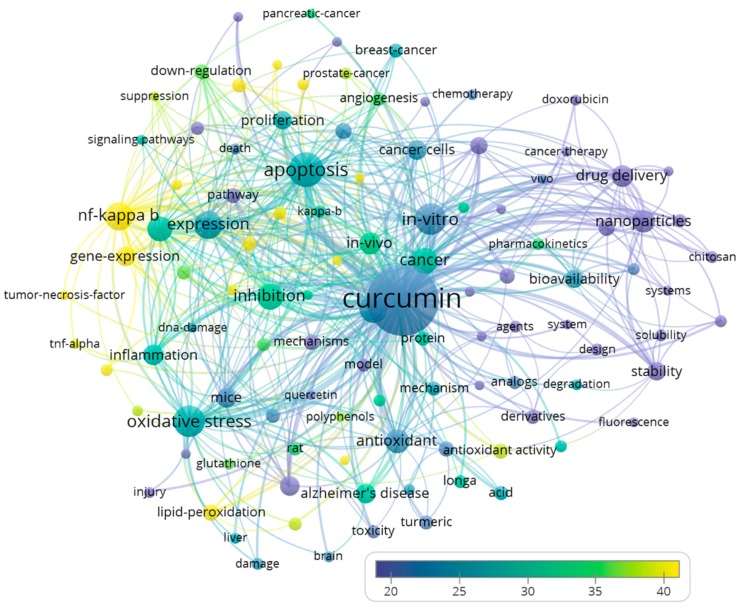
Bubble map visualizing keywords of the 18,036 curcumin publications. We used VOSviewer software to analyze and visualize recurring keywords added to the publications by the authors and by Web of Science. Only keywords that appeared in at least 1.0% (*n* = 181) of the publications were analyzed and visualized. There were 108 keywords that appeared in at least 1.0% of the evaluated publications. The word size indicates the appearance frequency of the words (multiple appearances in a single manuscript count as one). Two words are closer to each other if they co-occurred more frequently in the evaluated publications. Bubble colors represent the averaged citations of the terms.

Subsequently, we analyzed the keywords included into publications by authors and WoS (KeyWords Plus). As keywords are important for document searching and retrieval, authors usually carefully consider keyword selection for relevance, and a higher frequency of keywords use could indicate their importance. There were 108 keywords that appeared in at least 1.0% (*n* = 181) of the evaluated publications (Figure 2). In the bubble map presented as Figure 2, the size of the text/bubble is reflecting the frequency with which the keywords were used, and the color of the bubble is reflecting the citation frequency of the manuscripts in which the keywords were occurring. Therefore, a bigger size of the text/bubble might indicate a higher number of papers dealing with the respective topic, and a higher “intensity” of the color (according to the presented “color scale”) reflects higher impact (more citations obtained) of the manuscripts. The major themes were similar as reported above (Figure 1) for the words in the titles and abstracts of the 18,036 publications. Here, as evident from Figure 2, we observed that several cancers were frequently mentioned, such as breast (*n* = 417, citations per publication = 26.4), colon (*n* = 208, citations per publication = 44.9), colorectal (*n* = 271, citations per publication = 40.1), pancreatic (*n* = 207, citations per publication = 33.2), and prostate (*n* = 276, citations per publication = 37.5) cancers. Frequently mentioned components for the potential mechanisms included nuclear factor kappa-light-chain-enhancer of activated B cells (NF-κB, *n* = 1558, citations per publication = 48.4), nitric oxide synthase (NOS, *n* = 275, citations per publication = 73.9), and TNF-α (*n* = 203, citations per publication = 38.7). Curcumin may inhibit tumor growth by inhibiting NF-κB activation [34], and NOS [35]. Meanwhile, Alzheimer’s disease was also frequently mentioned (*n* = 746, citations per publication = 30.5). The top 20 recurring keywords are listed in Table 3. The keywords suggested that many of the studies were in vitro or in vivo using (cancer) cells, rats, and mice, but not clinical studies in humans.

**Figure 3:**
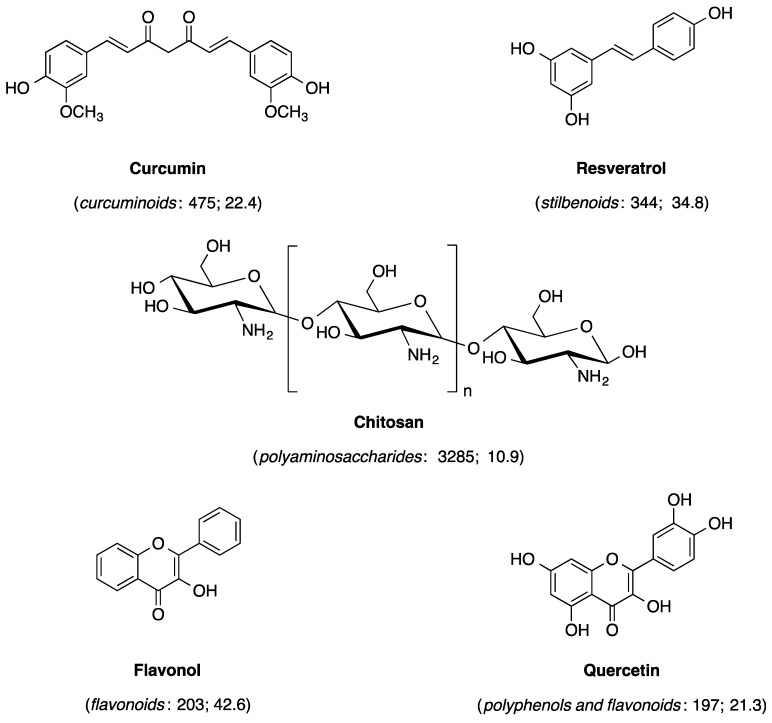
Chemical structures of key single chemicals or representatives of chemical classes that were often discussed in the evaluated curcumin publications. The cited compound classes (*italic*), number of publications and citations per publication for each chemical or representative chemical class are given in brackets.

Chemicals that were frequently mentioned in the keywords of evaluated curcumin publications included, in descending order, curcuminoids (*n* = 475, citations per publication = 22.4), resveratrol (*n* = 344, citations per publication = 34.8), chitosan (*n* = 285, citations per publication = 10.9), flavonoids (*n* = 203, citations per publication = 42.6), quercetin (*n* = 197, citations per publication = 21.3), and polyphenols (*n* = 192, citations per publication = 36.8) (Figure 3). In particular, chitosan was outlined as a useful carrier to deliver curcumin to target cells or sites in the form of nanoparticles [36,37].

To analyze the temporal changes in the use of keywords, we separated and assessed publications in four periods: 1989 and before, 1990s, 2000s, and 2010s. Unexpectedly, WoS did not record any keywords for the papers published in 1989 and before. The top 20 recurring keywords for each of the remaining three periods are listed in Table 4. It could be observed that antioxidant effects and cancer remained popular throughout the three periods. Drug delivery, bioavailability, and nanoparticles were emerging keywords that became popular since the 2010s, implying that more attention has been given to improve the delivery of curcumin to target sites.

As over half of the publications were published since 2014, we further analyzed the top 20 keywords used in publications contributed by the top five countries/regions (China, India, the United States, Iran, and Italy) since 2014 (Table 5). It seemed that the United States and Italy produced more curcumin-related manuscripts with relevance for Alzheimer’s disease. Manuscripts from Iran and Italy more frequently referred curcumin-related placebo-controlled and randomized controlled trials. These five countries quite commonly shared the other top keywords.

**Figure 4:**
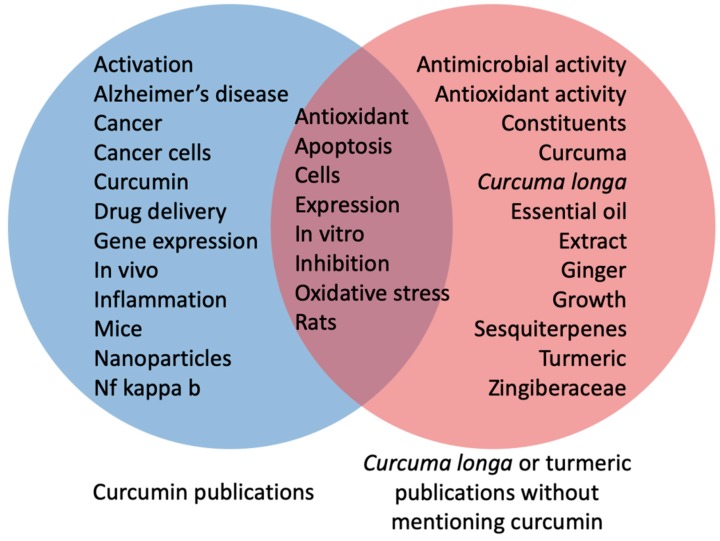
Venn diagram comparing the keywords used by curcumin-focused publications and those used by publications mentioning *Curcuma longa* or turmeric but not curcumin. Keywords from the former were more clinically relevant, whereas those from the latter were more related to plant science studies; commonly used keywords are presented in the middle of the diagram.

Since research focused on extracts of the plant *Curcuma longa*/turmeric is highly relevant and closely related to curcumin-focused research, an additional analysis was performed to evaluate the publications that mentioned *Curcuma longa* or turmeric without mentioning curcumin. A search was done with the strategy: TOPIC=(“curcum*” OR “tumeric*” OR “turmeric*” NOT “curcumin*”). We found 3920 publications resulting from this search. The most productive countries were India (*n* = 1041), China (*n* = 557), the United States (*n* = 388), Thailand (*n* = 286), and Japan (*n* = 265). The top five WoS categories were pharmacology and pharmacy (*n* = 720), food science technology (*n* = 561), plant sciences (*n* = 523), medicinal chemistry (*n* = 481), and integrative complementary medicine (*n* = 263). The top 20 keywords from these 3920 publications are listed in Table 6. Keywords from these publications were more related to plant science studies, concerning the constituents and extracts, as well as the antimicrobial and antioxidant activity. As expected, there was some overlapping with the curcumin-focused publications, reflected by common keywords such as apoptosis, inhibition, and oxidative stress (Figure 4).

## 4. Discussion

The current study analyzed the curcumin literature with a bibliometric approach. The increased publication shares from Asian countries in recent years were similarly observed for antioxidants-related literature [4]. Meanwhile, the huge contributions of the United States, China, and India were consistent with their dominance in ethnopharmacology research [20]. According to a previous bibliometric study, Curcuma papers oriented to nutraceuticals and functional foods research fields received large contributions from the UK and European states [23]. The unique geographic distribution here, different from bibliometric studies of other scientific disciplines, was the relatively large contribution from authors affiliated to Japanese and Korean institutions, especially on its biochemical and therapeutic properties, such as chemopreventive and anti-amyloidogenic effects [8,38].

Because clinical trials comprised of a small proportion of the curcumin publications analyzed in the current study, the bubble maps did not show many terms indicative of clinical studies. This is unlike the situation in neuroscience, where bubble maps clearly showed two clusters of terms, with one cluster being more related to cellular, molecular, and genetic aspects, and the other one being more related to clinical aspects [39].

By analyzing the studies describing clinical trial studies of curcumin, we found that oral curcumin (C3 complex) was a popular theme. Indeed, the C3 complex used in these studies is a standardized extract of dried rhizomes of *C. longa* and it represents the most clinically studied form of curcumin, evaluated in clinical trials in the context of cancer [32,33], but also in multiple other conditions, including Alzheimer’s disease [40], psoriasis vulgaris [41], oral lichen planus [42], osteoarthritis [43], inflammation associated with metabolic syndrome [44] and obesity [45], radiation dermatitis [46], as well as for modulation of human gut microbiome [47]. Moreover, while the low bioavailability of ingested curcumin had initially limited its clinical usage, a variety of different formulations with enhanced bioavailability have been recently developed and already studied in multiple clinical trials [48,49,50].

We did not analyze the authorship of the curcumin publications. This was because there existed many Chinese authors who have similar initials that caused inaccurate counting. For instance, according to the data downloaded from WoS, the most prolific author for the evaluated curcumin publications was Li Y., which represented Li Yan, Li Yu, and Li Yiwei upon closer examination. Analyzing authorship by considering authors’ full names was also not viable, because some publication records only listed author initials.

This study had some limitations, such as the use of a single database (WoS) to collect publications and their bibliometric data. Additionally, the analysis is of retrospective nature, and due to the fact that very recent trends (which still did not yield significant number of publications, due to their novelty) might remain undetected. Moreover, the readers should be reminded that articles mentioning *Curcuma longa* or turmeric/tumeric without mentioning curcumin were included in this work as a separate analysis, presented in Table 6 and Figure 4.

## 5. Conclusions

To conclude, a bibliometric analysis was performed to assess publications on curcumin research. Our findings have revealed that the United States and Asian countries, such as China, India, Japan, and South Korea, were major contributors. Most of the publications were focused on biochemistry, chemistry, oncology, and pharmacology. Over half of the publications were published since 2014, which mainly focused on the effects of curcumin against cancer, inflammation, and oxidative stress. Frequently investigated cancer types were breast, colon, colorectal, pancreatic, and prostate cancers. The large number of publications since 2014 could be attributed to the increased productivity of China (publications since 2014 = 2443; 68.9% of total contributions) and India (publications since 2014 = 1620; 51.8% of total contributions). Drug delivery, bioavailability, and nanoparticles have emerged as research themes for curcumin research. We expect that future studies should continue to improve delivery or even find new ways to deliver curcumin to target sites, so that more clinical studies can be supported.

## Figures and Tables

**Table 1:** The top five contributor journals, organizations, and countries/territories of the 18,036 manuscripts.

Contributor	Publication Count (% of Total)	Citation Per Manuscript
*Journal*		
PLOS One	234 (1.3%)	21.6
FASEB Journal	197 (1.1%)	4.3
Cancer Research	191 (1.1%)	39.0
RSC Advances	191 (1.1%)	7.6
Journal of Agricultural and Food Chemistry	187 (1.0%)	41.2
*Organization*		
Council of Scientific Industrial Research (CSIR India)	503 (2.8%)	12.1
University of Texas	307 (1.7%)	173.5
University of California	239 (1.3%)	71.1
Wenzhou Medical University	216 (1.2%)	9.6
Indian Institute of Technology	200 (1.1%)	20.2
*Country/Territory*		
United States	4073 (22.3%)	39.0
China	3546 (19.7%)	15.3
India	3128 (17.3%)	23.4
Japan	990 (5.5%)	28.7
South Korea	884 (4.9%)	24.3

Pearson’s correlation tests revealed that there was a significant positive correlation between total publication count and averaged citations per manuscript for affiliations (r = 0.147, *p* = 0.005), but not for countries/regions (r = 0.131, *p* = 0.333), or journals (r = 0.032, *p* = 0.656). These results implied that the citation advantage by publishing more only existed in the affiliation level.

**Table 2:** The top 20 recurring terms from titles and abstracts.

Term	Occurrence (% of 18,036 Publications)
Curcumin	13,722 (76.1%)
Effect	7958 (44.1%)
Study	7418 (41.1%)
Cell	6096 (33.8%)
Activity	5832 (32.3%)
Treatment	4917 (27.3%)
Compound	3661 (20.3%)
Level	3612 (20.0%)
Expression	3363 (18.6%)
Agent	3287 (18.2%)
Mechanism	3087 (17.1%)
Property	2939 (16.3%)
Concentration	2858 (15.8%)
Analysis	2735 (15.2%)
Inhibition	2720 (15.1%)
Pathway	2712 (15.0%)
Drug	2663 (14.8%)
Disease	2661 (14.8%)
Group	2606 (14.4%)
Protein	2597 (14.4%)

**Table 3:** The top 20 recurring keywords.

Keyword	Occurrence (% of 18,036 Publications)
Curcumin	9539 (52.9%)
Apoptosis	2223 (12.3%)
In vitro	1909 (10.6%)
Oxidative stress	1834 (10.2%)
Nf kappa b	1558 (8.6%)
Expression	1543 (8.6%)
Cells	1480 (8.2%)
Cancer	1290 (7.2%)
Inhibition	1269 (7.0%)
Activation	1230 (6.8%)
Antioxidant	1108 (6.1%)
Nanoparticles	1021 (5.7%)
Drug delivery	939 (5.2%)
In vivo	880 (4.9%)
Inflammation	808 (4.5%)
Mice	773 (4.3%)
Cancer cells	768 (4.3%)
Gene expression	768 (4.3%)
Alzheimer’s disease	746 (4.1%)
Rats	708 (3.9%)

**Table 4:** The top 20 recurring keywords in each decade.

1990s	Occurrence (% of 607)	2000s	Occurrence (% of 3683)	2010s	Occurrence (% of 13,636)
Curcumin	140 (23.1%)	Curcumin	1490 (40.5%)	Curcumin	7909 (58.0%)
Inhibition	37 (6.1%)	Apoptosis	442 (12.0%)	Apoptosis	1773 (13.0%)
Acid	33 (5.4%)	Nf kappa b	383 (10.4%)	In vitro	1688 (12.4%)
Tumor promotion	30 (4.9%)	Inhibition	355 (9.6%)	Oxidative stress	1538 (11.3%)
Activation	28 (4.6%)	Expression	306 (8.3%)	Expression	1228 (9.0%)
Chemoprevention	28 (4.6%)	Activation	298 (8.1%)	Cells	1188 (8.7%)
Dietary curcumin	26 (4.3%)	Oxidative stress	291 (7.9%)	Cancer	1038 (7.6%)
Mouse skin	24 (4.0%)	Cells	277 (7.5%)	Nanoparticles	1009 (7.4%)
Cancer	23 (3.8%)	Gene expression	267 (7.2%)	Nf kappa b	987 (7.2%)
Antioxidants	22 (3.6%)	Cancer	229 (6.2%)	Activation	904 (6.6%)
Curcuminoids	22 (3.6%)	Dietary curcumin	224 (6.1%)	Antioxidant	889 (6.5%)
Lipid peroxidation	22 (3.6%)	In vitro	219 (5.9%)	Inhibition	877 (6.4%)
Carcinogenesis	21 (3.5%)	Antioxidant	202 (5.5%)	Drug delivery	743 (5.4%)
Turmeric	20 (3.3%)	Lipid peroxidation	172 (4.7%)	In vivo	740 (5.4%)
Colon carcinogenesis	19 (3.1%)	Induction	147 (4.0%)	Inflammation	706 (5.2%)
Induction	19 (3.1%)	In vivo	138 (3.7%)	Mice	644 (4.7%)
In vitro	16 (2.6%)	Proliferation	133 (3.6%)	Bioavailability	610 (4.5%)
Protein-kinase-c	16 (2.6%)	Curcuminoids	125 (3.4%)	Stability	609 (4.5%)
Cells	15 (2.5%)	Mice	116 (3.1%)	Rats	588 (4.3%)
Gene expression	14 (2.3%)	Chemoprevention	114 (3.1%)	Therapy	540 (4.0%)

**Table 5:** The top 20 recurring keywords, in descending order, used in the publications contributed by the top five most productive countries since 2014.

(1) China	(2) India	(3) USA	(4) Iran	(5) Italy
Curcumin	Curcumin	Curcumin	Curcumin	Curcumin
Apoptosis	In vitro	In vitro	Oxidative stress	In vitro
In vitro	Nanoparticles	Apoptosis	In vitro	Oxidative stress
Expression	Apoptosis	Nf kappa b	Cancer	Nanoparticles
Nanoparticles	Oxidative stress	Expression	Nanoparticles	Nf kappa b
Oxidative stress	Cancer	Oxidative stress	Apoptosis	Apoptosis
Cells	Drug delivery	Cancer	Cells	Drug delivery
Activation	Cells	Nanoparticles	Placebo-controlled trial	Inflammation
Drug delivery	Antioxidant	Cells	Drug delivery	Cancer
Inhibition	Bioavailability	In vivo	Antioxidant	Expression
Cancer	Delivery	Inflammation	Inhibition	Alzheimer’s disease
Mice	Inhibition	Activation	Nf kappa b	Cells
Inflammation	Expression	Bioavailability	Randomized controlled trial	Randomized controlled trial
Stability	Stability	Inhibition	Double-blind	Placebo-controlled trial
Proliferation	Derivatives	Drug delivery	Inflammation	Polyphenols
In vivo	Cytotoxicity	Delivery	Therapy	Resveratrol
Nf kappa b	Nf kappa b	Stability	Quality of life	Therapy
Antioxidant	Design	Antioxidant	Delivery	In vivo
Delivery	Inflammation	Alzheimer’s disease	Expression	Double-blind
Therapy	Formulation	Mice	Mice	Activation

**Table 6:** The top 20 recurring keywords from publications mentioning *C. longa* or turmeric but not curcumin.

Keyword	Occurrence (% of 3920 Publications)
Turmeric	299 (7.6%)
*Curcuma longa*	231 (5.9%)
Essential oil	211 (5.4%)
In vitro	205 (5.2%)
Zingiberaceae	198 (5.1%)
Apoptosis	178 (4.5%)
Antioxidant	176 (4.5%)
Antioxidant activity	142 (3.6%)
Constituents	140 (3.6%)
Oxidative stress	131 (3.3%)
Extract	127 (3.2%)
Expression	126 (3.2%)
Antimicrobial activity	118 (3.0%)
Cells	114 (2.9%)
Growth	112 (2.9%)
Curcuma	110 (2.8%)
Inhibition	110 (2.8%)
Rats	102 (2.6%)
Sesquiterpenes	100 (2.6%)
Ginger	93 (2.4%)
